# Long-Term Cardiovascular and Mortality Risk in Patients with Pre-Existing Arrhythmia Post-SARS-CoV-2 Infection

**DOI:** 10.3390/diagnostics16010038

**Published:** 2025-12-22

**Authors:** Suhani Pahuja, Roham Hadidchi, Janhavi Tonge, Sonya Henry, Tim Q. Duong

**Affiliations:** Department of Radiology, Albert Einstein College of Medicine and Montefiore Health System, Bronx, NY 10647, USA; suhani.pahuja@einsteinmed.edu (S.P.); roham.hadidchi@einsteinmed.edu (R.H.); sonya.henry@einsteinmed.edu (S.H.)

**Keywords:** long COVID, post-acute sequelae of SARS-CoV-2 infection (PASC), ischemic heart disease, heart failure, socioeconomic status, social determinants of health

## Abstract

**Background/Objectives:** Individuals with arrhythmia who survived COVID-19 could be susceptible to long-term cardiovascular complications and clinical outcomes. **Methods:** We performed a retrospective cohort study of adults with a history of arrhythmia in the Montefiore Health System (1 January 2016–17 August 2024). COVID-19 status was determined by a positive or negative polymerase-chain-reaction test. Outcomes included all-cause mortality, first-time myocardial infarction (MI), heart failure (HF), ischemic or hemorrhagic stroke, and major adverse cardiovascular events (MACE: defined as MI, HF, stroke, or death) > 30 days post-index date. Cox proportional hazards and Fine–Gray competing risk models, adjusted for demographic, clinical, socioeconomic, and COVID-19 vaccination variables, were employed. The association of outcomes with blood biomarkers taken at time of infection were also assessed in hospitalized COVID-19 patients. **Results:** Among the 6830 arrhythmia patients, 985 were hospitalized for COVID-19, 1591 were not hospitalized for COVID-19, and 4254 did not have COVID-19. Patients hospitalized for COVID-19 had a higher risk of all-cause mortality (adjusted hazard ratio = 2.90, 95% confidence-interval [2.08, 4.04]), first-time MI, HF, and MACE compared to controls without COVID-19. No increased risk was observed among non-hospitalized COVID-19-positive patients compared to controls, except for all-cause mortality. Older age, male sex, Medicaid, and significant comorbidities were associated with the risk of MACE. Elevated levels of creatinine, lactate dehydrogenase, D-dimer, neutrophil-to-lymphocyte ratio, low hemoglobin, and low left ventricular ejection fraction during infection were associated with higher future MACE risk. **Conclusions.** In individuals with arrhythmia, severe COVID-19 is associated with increased long-term risks of mortality and new-onset cardiovascular complications, while mild infection with mortality risk. These findings highlight the need for long-term cardiovascular monitoring in this population.

## 1. Introduction

Cardiac arrhythmias affect over 6 million adults in the United States, contributing to more than 450,000 deaths each year [1,2]. People with these arrhythmias face a significantly higher risk of developing myocardial infarction (MI), heart failure (HF), and stroke [3,4]. Although the morbidity and mortality associated with cardiac arrhythmias are well documented, the emergence of COVID-19 has added new challenges to the long-term management of this high-risk group [5]. Some studies have shown that COVID-19 survivors are at increased risk of incident MI, HF, stroke, and arrhythmia after infection. However, no research has examined whether SARS-CoV-2 infection adversely affects long-term cardiovascular outcomes in patients with pre-existing cardiac arrhythmias [5,6,7,8,9].

SARS-CoV-2 infection may worsen cardiovascular risk in individuals with pre-existing arrhythmias. Viral entry via angiotensin-converting enzyme-2 receptor can injure cardiomyocytes and vascular endothelium and promote apoptosis, vascular dysfunction, and thrombosis, while heightened sympathetic activation and autonomic imbalance can exacerbate arrhythmias even after acute recovery [10]. Severe COVID-19-related hypoxemia, electrolyte derangements, and fluid shifts further destabilize cardiac rhythm and increase the risk of heart failure or ischemic events [11,12,13]. Endothelial injury and microvascular thrombosis may also impair myocardial perfusion, elevating the risk of MI and stroke [12]. Together, these pathways suggest heightened vulnerability among patients with baseline arrhythmias. Given the high global burden of arrhythmia and the widespread reach of COVID-19, clarifying their interaction is an important public health priority.

In this study, we investigated the incidence of new-onset cardiovascular outcomes over a follow-up period of 4.5 years post-COVID-19 in arrhythmic patients in the Montefiore Health System, a large urban health system in the Bronx, New York. This setting is racially and ethnically diverse, containing a large low-income population, which resulted in a disproportionately high COVID-19 burden during multiple waves of the pandemic. In addition to evaluating overall incidence, the effects of demographic and socioeconomic factors, vaccination status, blood-based biomarkers, and unmet social needs on the risk for post-COVID-19 MACE outcomes were also determined. By combining long-term follow-up with clinical diagnoses and granular health system data, this study contributes to the current understanding of post-COVID-19 cardiovascular effects in arrhythmic patients.

## 2. Materials and Methods

### 2.1. Data Sources

This retrospective cohort study was approved by the Einstein–Montefiore Institutional Review Board (#2021–13658). Electronic health records (EHRs) were extracted as previously described [13,14,15,16,17,18,19,20] from 1 January 2016 to 17 August 2024 in the Montefiore Health System, which includes multiple hospitals and outpatient clinics in the Bronx and surrounding areas. To ensure data quality, we routinely reviewed charts of relevant EMR for subsets of patients. Recognizing the inherent limitations of retrospective designs, including potential biases, confounding factors, and competing risks, multiple statistical approaches and sensitivity analyses were used to enhance internal validity and support the findings. Given the size and diversity of our patient population, these results are likely to be widely applicable, although additional studies with external cohorts are needed for broader generalization.

### 2.2. Study Cohort

Only adults (≥21 years old) with cardiac arrhythmia at index date but without prior history of outcomes (MI, HF, or stroke) were included. All conditions were defined by ICD-10 codes, listed in Supplementary Table S1. Patients classified as COVID-19+ were those who tested positive by polymerase chain reaction (PCR), and the index date was defined as the date of the first positive test. COVID-19 controls were those who never tested positive, and the index date was defined as the date of the first negative test. Hospitalization status was associated with the COVID-19 visit (within 2 weeks of PCR test). We excluded patients who did not return to our health system 30 days or more after the index date and those who experienced MACE events or death within 30 days after the index date. These exclusions were applied to both the COVID-19+ and COVID-19− groups to reduce the misclassification of acute-phase events that may not represent long-term cardiovascular risk. However, this might introduce selection bias by removing sicker individuals; thus, a sensitivity analysis without these 30-day exclusions was also performed to evaluate for this potential bias.

### 2.3. Variables

Demographic data analyzed included age at the index date, sex, race, and ethnicity. Pre-existing comorbidities considered were coronary artery disease (CAD), hypertension (HTN), type 2 diabetes (T2DM), chronic obstructive pulmonary disease (COPD), asthma, chronic kidney disease (CKD), liver disease, obesity, and tobacco use, all defined using ICD-10 codes at the index date.

Data was collected on COVID-19 vaccination, and patients with at least one vaccination before the index date were considered vaccinated. Vaccination information came from the New York State Immunization Information System (including New York City data), patient self-report, Care Everywhere data shared across EPIC organizations, and vaccinations given within the Montefiore Health System.

Socioeconomic data were collected, including the median annual household income for the Zone Improvement Plan (ZIP) code, insurance status, and unmet social needs. Insurance type was categorized as private, Medicare, Medicaid, or uninsured, and information was obtained from the EHR. The voluntary unmet social needs screener included nine categories: housing, food insecurity, utilities, health, transportation, medications, child or elderly care, legal services, and family safety. Patients were classified into three groups: at least one unmet social need, no unmet social needs, and unknown.

Stratification of COVID-19-positive patients during the acute phases of infection enabled us to evaluate the severity of SARS-CoV-2. The following biomarkers were collected at the time of infection from the hospitalized COVID-19+ cohort: neutrophil-to-lymphocyte ratio (NLR), ferritin (μg/L), D-dimer (μg/mL), creatinine (mg/dL), hemoglobin (g/dL), aspartate aminotransferase (U/L), C-reactive protein (CRP, mg/dL), B-type natriuretic peptide (pg/mL), lactate dehydrogenase (U/L), left ventricular ejection fraction (LVEF, %), troponin I, and troponin T (ng/mL). LVEF was measured by echocardiogram and obtained during acute COVID-19.

### 2.4. Outcome Events

All-cause mortality, first-time MI, HF, stroke, and MACE (a composite of these four outcomes) recorded in the EHR over 30 days and up to 4.5 years after the index date were considered outcome events. Follow-up time was calculated as the period from the index date to the first diagnosis date for patients who experienced the outcome or until the date of death or last recorded visit for those who did not. The cut-off date was 17 August 2024.

### 2.5. Statistical Analysis

Data processing and statistical analysis were performed using Python (version 3.8.19) and R (version 4.4.0), and *p* values less than 0.05 were considered statistically significant. The χ^2^ test was used to compare categorical variables between groups, and the independent *t* test was employed for comparing continuous variables. To assess the magnitude of differences between groups, we also calculated standardized mean differences (SMD), considering values greater than 0.10 as potentially meaningful. Cox proportional hazards models evaluated the risk of outcomes for all-cause mortality and MACE, while Fine–Gray sub-distribution competing risk regression models were used for MI, HF, and ischemic or hemorrhagic stroke to account for mortality as a competing risk. Baseline adjustments for age, sex, race, ethnicity, comorbidities, insurance status, tertile of ZIP code median income, presence of unmet social needs, and SARS-CoV-2 vaccination status were performed using multivariate regression analysis. Vaccination status was modeled as a time-fixed variable, and all covariates were measured at index date. To create a pseudo-population for analysis, inverse probability weighting (IPW) was employed based on the same covariates that were adjusted for in multivariate analysis. Outcomes were also analyzed using Kaplan–Meier and cumulative incidence curves. For COVID-19 hospitalized patients with blood biomarkers collected at admission, these biomarkers were examined in relation to the risk of MACE. No imputation was used.

### 2.6. Sensitivity Analyses

Patients who were lost to follow-up or experienced MACE outcomes within 30 days of the index date were excluded to prevent bias from survivor effects. These exclusions were not applied in sensitivity analyses. No evidence of violation of the proportional hazard assumption was found using Schoenfeld residuals or when evaluating time-by-covariate interaction terms. Additionally, a quantitative bias analysis for unmeasured confounding was also conducted.

## 3. Results

### 3.1. Cohort Selection

**Figure 1:**
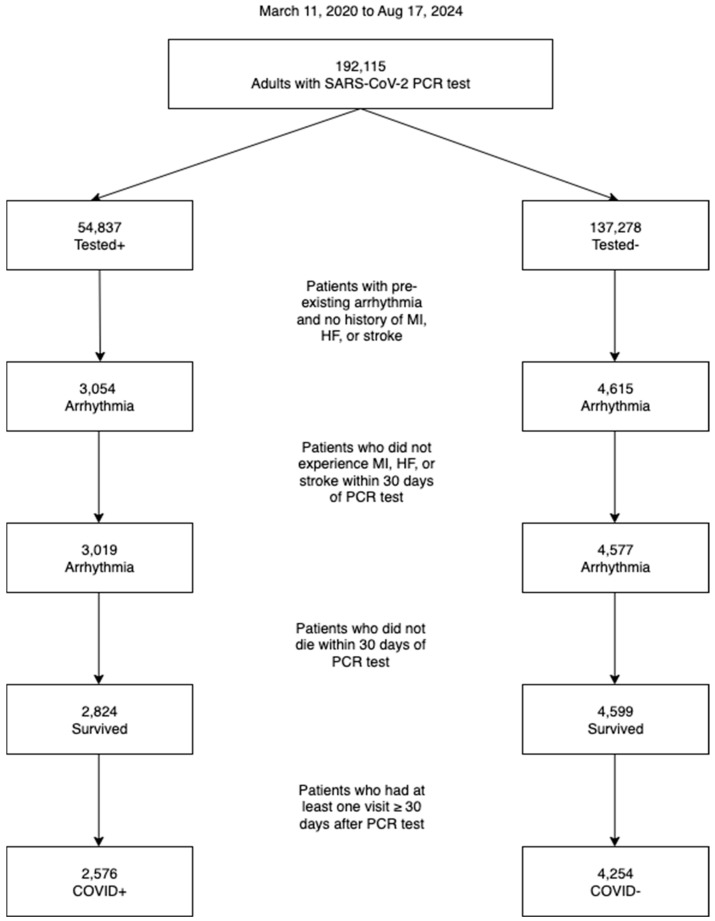
Patient selection flowchart. PCR, polymerase chain reaction. MI, myocardial infarction. HF, heart failure.

Figure 1 depicts the patient selection flowchart. From 11 March 2020 to 12 January 2024, there were 192,115 adults ≥21 years old who had a SARS-CoV-2 PCR test performed to determine the existence of respiratory infection. There were 3054 patients who were COVID-19+ and 4615 patients who were COVID-19− who had pre-existing arrhythmia but not history of MI, HF, or stroke. Of the COVID-19+ patients, 195 (6.46%) died during acute (within 30 days) infection. Upon excluding patients who died or developed a MACE outcome within the first 30 days of PCR test, 230 (7.53%) COVID-19+ and 16 (0.35%) COVID-19− patients were lost to follow-up within the first 30 days. After exclusion, there were 6830 arrhythmia patients, out of which 985 were hospitalized for COVID-19, 1591 were not hospitalized for COVID-19, and 4254 did not have COVID-19.

Table 1 presents the characteristics of patients with pre-existing arrhythmia who had no history of MI, HF, or stroke, comparing those with and without COVID-19 (unmatched cohort). COVID-19 patients were, on average, 3 years older (63.49 vs. 60.74 years, independent *t* test *p* < 0.005), less likely to be non-Hispanic White (12.69% vs. 14.83%, χ^2^ *p* = 0.015), more likely to be Hispanic (42.70% vs. 39.54%, χ^2^ *p* = 0.011), and more likely to have all pre-existing comorbidities (χ^2^ *p* < 0.005 for all), except for tobacco use. They were also less likely to be on private insurance (34.70% vs. 38.76%, χ^2^ *p* < 0.005), more likely to be on Medicare (32.84% vs. 27.43%, χ^2^ *p* < 0.005), and less likely to fall in the top-third income group (29.04% vs. 32.53%, χ^2^ *p* < 0.005).

### 3.2. Main Analysis

Cox proportional and Fine–Gray sub-distribution hazard ratios (HRs) for COVID-19 and the risk of cardiovascular outcomes are shown in Table 2. Compared to the COVID-19− group, patients hospitalized with COVID-19 were more likely to experience all-cause mortality, MI, HF, and MACE after adjusting for age, sex, race, ethnicity, pre-existing comorbidities, insurance, and median income of ZIP code. Supplementary Table S2 displays the full results of the multivariate models.

A weighted cohort was formed using IPW, and an analysis was conducted to confirm these findings. Cohorts based on hospitalized COVID-19 patients, non-hospitalized COVID-19 patients, and COVID-19− patients are detailed in Supplementary Table S3. Covariate adjustment with IPW produced similar results. The only outcomes significantly more common in the COVID-19+ hospitalized group compared to the COVID-19− group were MI and MACE, likely because patients not heavily weighted by IPW did not contribute as much data, which reduced statistical power. Multivariate regression and IPW indicated that hospitalized COVID-19 patients had a higher risk of all-cause mortality compared to the COVID-19− group. Additionally, patients on Medicaid showed increased risk of all-cause mortality, MI, and MACE.

**Figure 2:**
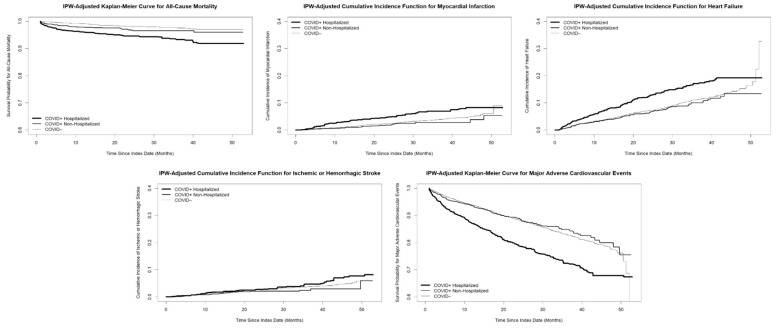
Kaplan–Meier (all-cause mortality and major adverse cardiovascular events) and cumulative incidence function (myocardial infarction, heart failure, and ischemic or hemorrhagic stroke) up to 52 months follow-up among COVID-19+ hospitalized, COVID-19+ non-hospitalized, and COVID-19− groups. The three groups were inverse probability weighting (IPW)-adjusted according to age, sex, race, ethnicity, comorbidities, insurance status, tertile of Zone Improvement Plan code median income, presence of unmet social needs, and SARS-CoV-2 vaccination status.

Figure 2 displays IPW-adjusted Kaplan–Meier and cumulative incidence curves for outcomes. Among all groups, COVID-19+ hospitalized patients experienced worse outcomes than COVID-19+ non-hospitalized and COVID-19− patients. The outcomes of the COVID-19+ non-hospitalized group were the most similar to those of the COVID-19− group.

MACE was assessed using biomarkers collected during acute COVID-19 to explore potential links between outcomes and these biomarkers. Multivariate Cox proportional adjusted HRs for biomarkers at the time of infection and the risk of MACE in hospitalized COVID-19 patients are shown in Table 3. Creatinine ≥ 1.1 mg/dL (adjusted HR = 1.47 [1.07, 2.01]), lactate dehydrogenase ≥ 400 U/L (adjusted HR = 1.41 [1.01, 1.97]), D-dimer ≥ 1.5 μg/mL (adjusted HR = 1.45 [1.07, 1.94]), hemoglobin ≤ 9.2 g/dL (adjusted HR = 2.03 [1.53, 2.69]), NLR ≥ 10 (adjusted HR = 1.29 [1.00, 1.66]), and LVEF < 50% (adjusted HR = 2.71 [1.40, 5.25]) were associated with an increased risk of MACE. Aspartate aminotransferase ≥ 100 U/L (adjusted HR = 1.05 [0.66, 1.67]), C-reactive protein ≥ 15 mg/dL (adjusted HR = 1.33 [0.96, 1.83]), ferritin ≥ 700 µg/L (adjusted HR = 1.09 [0.77, 1.53]), B-type natriuretic peptide ≥ 100 pg/mL (adjusted HR = 0.88 [0.47, 1.65]), and troponin I > 0.20 ng/mL or troponin T > 0.05 ng/mL (adjusted HR = 1.61 [0.79, 3.28]) were not associated with an increased risk of MACE.

A sensitivity analysis was conducted without excluding patients who died within 30 days of index date, as shown in Appendix S1. The results were consistent in direction, although patients with COVID-19 faced a significantly higher risk of all-cause mortality when patients who died acutely were not excluded.

**Figure 3:**
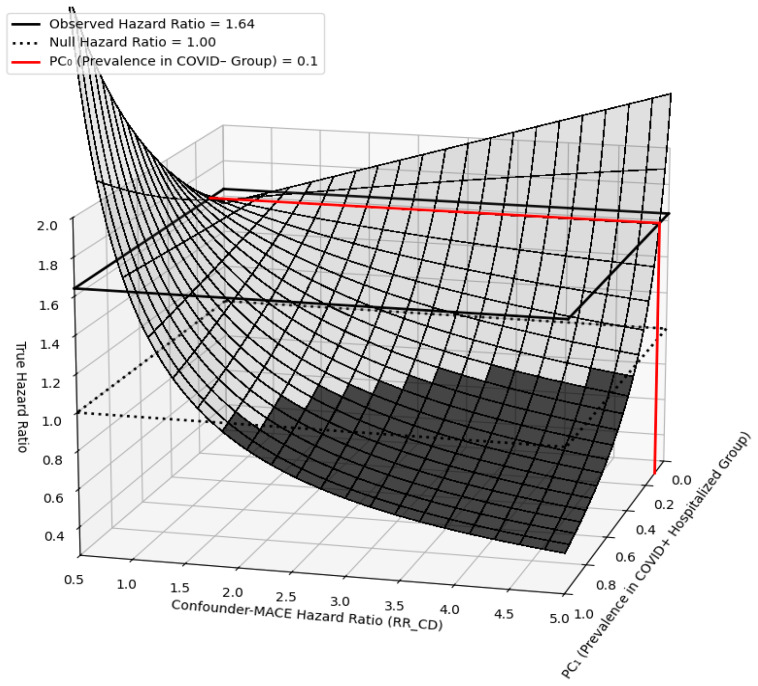
Sensitivity analysis evaluating the potential influence of residual confounding. The surface depicts the true hazard ratio, assuming an observed hazard ratio of 1.64 and a prevalence of the unmeasured confounder (PC_0_) of 0.1 (10%) in the COVID-19− group. Relative risk confounder–disease (RR_CD) represents the independent association between an unmeasured confounder and the risk of first-time major adverse cardiovascular events (MACE). If a confounder had an independent risk ratio of 1.80 for new-onset MACE and was at least 3.5 times more prevalent in the COVID-19+ hospitalized group compared to the COVID-19− group, then the observed effect could be nullified, which is unlikely. The darker shade indicates true hazard ratio less than one.

To assess the potential impact of residual confounding on our primary outcome, we performed another sensitivity analysis. An unmeasured confounder independently linked to MACE with an HR of 1.80 would need to be at least 3.5 times more common in the COVID-19+ hospitalized group compared to the COVID-19− group to fully account for the observed multivariate-adjusted hazard ratio of 1.64 (COVID-19+ hospitalized for MACE) (Figure 3).

## 4. Discussion

Our study represents the first effort to characterize the long-term cardiovascular risk profile of COVID-19 survivors with pre-existing arrhythmia over a follow-up period of up to 4.5 years post-infection and compared to arrhythmia patients without COVID-19. We found that (i) COVID-19+ hospitalized patients with pre-existing arrhythmia are at higher risk of first-time MI, HF, all-cause mortality, and MACE compared to COVID-19− controls after adjusting for demographic, clinical, and socioeconomic variables, as well as SARS-CoV-2 vaccination status; (ii) COVID-19+ non-hospitalized patients are at higher adjusted risk of all-cause mortality compared to COVID-19− controls but at similar risk of MI, HF, stroke, and MACE compared to COVID-19− controls; (iii) the risk of MACE was higher in those who were older, male, had comorbidities, and were on Medicaid; (iv) among hospitalized COVID-19+ patients, blood biomarkers at the time of infection (creatinine ≥ 1.1 mg/dL, LDH ≥ 400 U/L, D-dimer ≥ 1.5 µg/mL, hemoglobin ≤ 9.2 g/dL, NLR ≥ 10, and LVEF < 50%) were associated with future first-time MACE risk.

Several prior studies have reported an increased risk of first-time cardiovascular disorders, including MACE, arrhythmia, MI, HF, and stroke, among COVID-19 survivors compared to COVID-19− controls [21,22,23,24]. However, to our knowledge, no studies have specifically examined long-term post-COVID-19 cardiovascular outcomes in individuals with pre-existing arrhythmia. Patients with arrhythmia are at increased risk of post-COVID-19 cardiovascular complications, likely due to elevated baseline risk factors and unique pathophysiological features that COVID-19 may exacerbate [25,26,27]. The virus can directly invade cardiomyocytes via the angiotensin-converting enzyme-2 receptor, potentially disrupting myocardial electrical conduction [6,28,29]. The systemic inflammatory response also boosts sympathetic activity and may cause dysregulation of cardiac ion channels, further destabilizing electrical conduction [30,31]. Additionally, severe hypoxemia and electrolyte imbalances during acute infection disrupt cellular ion gradients, alter action potential propagation, and promote arrhythmogenic remodeling in cardiac tissue [32,33,34]. As a result, individuals with pre-existing arrhythmias might be particularly vulnerable to further disruptions in cardiac electrophysiology after COVID-19. Notably, low LVEF during acute infection predicted a higher subsequent risk of MACE. This finding supports the idea that myocardial injury and electrical dysfunction during SARS-CoV-2 infection could contribute to the increased long-term cardiovascular risk in this vulnerable group.

Previous studies have examined long-term cardiovascular outcomes in patients with pre-existing coronary artery disease, concluding that hospitalized COVID-19 patients are at higher risk of MACE outcomes [35]. Other studies have examined those with pre-existing diabetes (type 1 and type 2) [36,37], chronic kidney disease, [38,39] chronic obstructive pulmonary disease [40] and rheumatoid arthritis [41]. This study specifically looked at COVID-19 patients with pre-existing arrhythmia (both atrial and ventricular) and incidence of long-term cardiovascular outcomes.

Overall, the findings support the idea that severe COVID-19 can initiate a chain reaction of inflammation and vascular damage with long-lasting cardiovascular effects, especially in those with existing arrhythmia. Recognizing hospitalized patients with abnormal biomarker levels can help in risk assessment after recovery and guide more targeted follow-up and prevention efforts.

Clinically, this study highlights the importance of ongoing cardiovascular monitoring not only in high-risk or hospitalized patients but also in those with milder COVID-19 cases. Public health strategies should, therefore, consider this broader at-risk group when planning follow-up care and preventive measures. Long-term cardiovascular monitoring for post-COVID-19 patients with arrhythmias should follow a structured and proactive approach that incorporates ambulatory rhythm monitoring, routine ECGs, and targeted cardiac imaging—such as echocardiography and cardiac MRI when inflammation or fibrosis is suspected and coronary angiography when clinically indicated. Selective biomarker testing can help evaluate new or worsening symptoms, while early electrophysiology referral is appropriate for symptomatic or high-burden arrhythmias. Risk-factor optimization, including management of hypertension, diabetes, dyslipidemia, obesity, and sleep apnea, remains central to reducing ongoing vulnerability. 

The risk of MACE was higher in individuals who were older; male; had comorbidities such as CAD, HTN, COPD, CKD, and tobacco use; and were on Medicaid. This aligns with the literature indicating that Medicaid enrollment serves as a proxy for lower socioeconomic status and limited healthcare access, which are associated with higher rates of undiagnosed or undertreated arrhythmias and increased cardiovascular risk [42,43]. Several studies also show that among those with ventricular arrhythmias, males face a higher cardiovascular risk, although the reasons for this are not fully understood [2]. These findings are consistent with previous research demonstrating that advanced age, along with cardiometabolic, pulmonary, and renal comorbidities, including CAD, HTN, COPD, CKD, and tobacco use, are strong predictors of poor prognosis in patients with baseline arrhythmia [44,45,46,47]. Our findings reinforce the established need for targeted secondary prevention in patients with arrhythmia, especially those who are older, have multiple chronic conditions, or show signs of socioeconomic vulnerability [48,49,50,51,52]. Customized interventions, such as intensified pharmacologic management, cardiac rehabilitation, remote monitoring, and social support services, may be necessary to lower the recurrence of cardiovascular events in these high-risk groups after COVID-19. These data also emphasize the importance of including social and clinical risk factors in arrhythmia care models, particularly in the face of emerging threats like COVID-19, which may impact the most vulnerable patients differently.

In order to estimate the role of unmeasured confounding in the association between COVID-19 hospitalization and the development of MACE outcomes, a quantitative bias analysis was conducted. It was found that an unmeasured confounder with a risk ratio of 1.80 for new-onset MACE would need to be at least 3.5 times more common in the COVID-19+ hospitalized group than in the COVID-19− group—which is highly unlikely—to reduce the hazard ratio for MACE in the COVID-19+ hospitalized group to the null.

### Limitations

There are several limitations in this study. This study depended on the accuracy of the EHR, which could contain documentation errors. Antibody tests and at-home COVID-19 tests were not consistently recorded and, therefore, were not used as indicators of COVID-19 status. Misclassification of patients as COVID-19+ was possible, likely leading to an underestimation of the infection’s impact on outcomes. The contribution of acute biomarkers to outcomes in non-hospitalized COVID-19 patients was not examined, as laboratory tests were usually not clinically indicated. Our findings may not be fully representative of less diverse populations. There could be potential selection bias due to the exclusion of patients who were lost to follow-up within 30 days of the index date and those who experienced MACE events or death within 30 days after the index date. Although this was done to minimize acute-phase confounding and clarify long-term risk, it may have excluded patients at the highest baseline risk, thereby underestimating the true link between COVID-19 and MACE. Our cohort is diverse, and our findings may not be generalizable to less diverse populations. Medication prescription and compliance data are difficult to obtain and incompletely documented in EMR databases. We, therefore, did not assess the effects of medications. We relied on ZIP code median income, which may not always reflect individual income. COVID-19 patients may have had more close follow-ups. We chose to dichotomize normal and abnormal states and did not analyze laboratory data as continuous variables because these variables are very heterogeneous and, along with the large number of variables, could result in overfitting.

Our sensitivity analysis without this exclusion showed similar results. All major confounders were adjusted for through multivariate regression and IPW, and only an extreme residual confounding scenario could entirely explain the observed effect size. Despite our efforts, unintentional patient selection biases and other unmeasured biases cannot be fully excluded in any observational study.

## 5. Conclusions

Our findings emphasize the significant and lasting cardiovascular risks faced by COVID-19 survivors with pre-existing arrhythmias. Hospitalized patients showed notably higher risk for first-time myocardial infarction, heart failure, all-cause mortality, and MACE, even after thorough adjustment for confounders. Although non-hospitalized patients also had increased mortality risk, they did not show higher risk for other specific cardiovascular outcomes. Importantly, blood-based biomarkers at the time of COVID-19 hospitalization were associated with future MACE, providing potential for early risk stratification. These results highlight the need for long-term cardiovascular monitoring and customized post-COVID-19 care for patients with arrhythmias, especially those with high-risk profiles.

## Figures and Tables

**Table 1:** Characteristics of patients with pre-existing arrhythmia but no history of myocardial infarction, heart failure, or stroke with and without COVID-19. SD, standard deviation. SMD, standardized mean difference.

	COVID-19+ (*n* = 2576)	COVID-19− (*n* = 4254)	*p* Value	SMD
Follow-Up Time (Months), mean ± SD	23.66 ± 13.79	28.81 ± 14.23	<0.005	0.37
Age at Index Date (Years), mean ± SD	63.49 ± 17.08	60.74 ± 16.63	<0.005	0.16
Female, n (%)	1544 (59.94%)	2506 (58.91%)	0.42	0.021
Race and Ethnicity, n (%)				
Non-Hispanic White	327 (12.69%)	631 (14.83%)	0.015	0.062
Black	890 (34.55%)	1481 (34.81%)	0.84	0.0056
Asian	87 (3.38%)	133 (3.13%)	0.62	0.014
Other Race	1272 (49.38%)	2009 (47.23%)	0.089	0.043
Hispanic	1100 (42.70%)	1682 (39.54%)	0.011	0.064
Pre-Existing Comorbidities, n (%)				
Arrhythmia	2576 (100.00%)	4254 (100.00%)		0
Atrial Fibrillation	715 (27.76%)	983 (23.11%)	<0.005	0.11
Atrial Flutter	141 (5.47%)	145 (3.41%)	<0.005	0.1
Conduction Disease	735 (28.53%)	1089 (25.60%)	0.0086	0.066
Ventricular Arrhythmia	145 (5.63%)	187 (4.40%)	0.025	0.057
Bradyarrhythmia	1001 (38.86%)	1728 (40.62%)	0.16	0.036
SVT	280 (10.87%)	331 (7.78%)	<0.005	0.11
Nonspecific/Misc Arrhythmia	431 (16.73%)	649 (15.26%)	0.11	0.04
Coronary Artery Disease	533 (20.69%)	659 (15.49%)	<0.005	0.14
Hypertension	1945 (75.50%)	2909 (68.38%)	<0.005	0.16
Type 2 Diabetes	1029 (39.95%)	1297 (30.49%)	<0.005	0.20
COPD	201 (7.80%)	189 (4.44%)	<0.005	0.14
Asthma	681 (26.44%)	906 (21.30%)	<0.005	0.12
Chronic Kidney Disease	696 (27.02%)	724 (17.02%)	<0.005	0.24
Liver Disease	375 (14.56%)	505 (11.87%)	<0.005	0.079
Obesity	1502 (58.31%)	2274 (53.46%)	<0.005	0.098
Tobacco Use	1067 (41.42%)	1751 (41.16%)	0.85	0.0053
Insurance, n (%)				
Medicaid	718 (27.87%)	1231 (28.94%)	0.36	0.024
Medicare	846 (32.84%)	1167 (27.43%)	<0.005	0.12
Private	894 (34.70%)	1649 (38.76%)	<0.005	0.084
Uninsured	118 (4.58%)	207 (4.87%)	0.63	0.013
Income Group, n (%)				
Lower Third	1025 (39.79%)	1636 (38.46%)	0.29	0.027
Middle Third	803 (31.17%)	1234 (29.01%)	0.062	0.047
Top Third	748 (29.04%)	1384 (32.53%)	<0.005	0.076
Unmet Social Needs, n (%)				
At Least One Unmet Social Need	269 (10.44%)	424 (9.97%)	0.56	0.016
No Unmet Social Needs	790 (30.67%)	1239 (29.13%)	0.19	0.034
Status Unknown	1517 (58.89%)	2591 (60.91%)	0.10	0.041
Hospitalized Due to COVID-19, n (%)	985 (38.24%)	0 (0.00%)	<0.005	1.10
Vaccinated for SARS-CoV-2, n (%)	1048 (40.68%)	1298 (30.51%)	<0.005	0.21
Outcomes, n (%)				
All-Cause Mortality	116 (4.50%)	78 (1.83%)	<0.005	0.15
Myocardial Infarction	93 (3.61%)	122 (2.87%)	0.10	0.042
Heart Failure	260 (10.09%)	356 (8.37%)	0.018	0.060
Ischemic or Hemorrhagic Stroke	74 (2.87%)	122 (2.87%)	1.00	0.00029
MACE	433 (16.81%)	553 (13.00%)	<0.005	0.11

**Table 2:** Cox proportional (all-cause mortality and major adverse cardiovascular events) and Fine–Gray sub-distribution (myocardial infarction, heart failure, and ischemic or hemorrhagic stroke) adjusted hazard ratios (HRs) for different outcomes grouped by COVID-19 status (COVID-19+ hospitalized and COVID-19+ non-hospitalized vs. COVID-19−). (**A**) Multivariate regression and (**B**) inverse probability weighting both adjusted for baseline age, sex, race, ethnicity, comorbidities, insurance status, tertile of Zone Improvement Plan code median income, presence of unmet social needs, and SARS-CoV-2 vaccination status. HR, hazard ratio. CI, confidence interval.

	**(A) Multivariate Regression**			
**Outcome**	**COVID-19+ Hospitalized vs. COVID-19−**		**COVID-19+ Non-Hospitalized vs. COVID-19−**	
	**Adjusted HR [95% CI]**	***p* Value**	**Adjusted HR [95% CI]**	***p* Value**
All-Cause Mortality	2.90 [2.08, 4.04]	<0.005	1.67 [1.13, 2.47]	0.010
Myocardial Infarction	1.57 [1.14, 2.17]	0.0060	0.82 [0.55, 1.23]	0.34
Heart Failure	1.51 [1.24, 1.84]	<0.005	0.94 [0.75, 1.18]	0.62
Stroke	1.24 [0.87, 1.78]	0.24	0.83 [0.55, 1.24]	0.36
MACE	1.64 [1.40, 1.91]	<0.005	0.99 [0.83, 1.18]	0.92
	**(B) Inverse Probability Weighting-Adjusted**			
**Outcome**	**COVID-19+ Hospitalized vs. COVID-19−**		**COVID-19+ Non-Hospitalized vs. COVID-19−**	
	**HR [95% CI]**	***p*** **Value**	**HR [95% CI]**	***p*** **Value**
All-Cause Mortality	3.02 [2.12, 4.30]	<0.005	1.65 [1.11, 2.45]	0.013
Myocardial Infarction	1.74 [1.21, 2.49]	<0.005	0.75 [0.49, 1.15]	0.18
Heart Failure	1.55 [1.24, 1.93]	<0.005	0.91 [0.73, 1.15]	0.45
Stroke	1.21 [0.83, 1.77]	0.32	0.75 [0.50, 1.14]	0.18
MACE	1.68 [1.40, 2.01]	<0.005	0.96 [0.80, 1.15]	0.66

**Table 3:** Cox proportional hazard ratios (HRs) for biomarkers at time of infection and risk of major adverse cardiovascular events among patients hospitalized for COVID-19 (*n* = 985). Inverse probability weighting (IPW) adjusted for baseline age, sex, race, ethnicity, comorbidities, insurance status, tertile of Zone Improvement Plan code median income, presence of unmet social needs, and SARS-CoV-2 vaccination status.

Biomarker Predictor	IPW-Adjusted HR [95% CI]	*p* Value
Aspartate Aminotransferase ≥ 100 U/L (n = 946)	1.05 [0.66, 1.67]	0.83
C-Reactive Protein ≥ 15 mg/dL (n = 708)	1.33 [0.96, 1.83]	0.084
Creatinine ≥ 1.1 mg/dL (n = 979)	1.47 [1.07, 2.01]	0.017
Lactate Dehydrogenase ≥ 400 U/L (n = 910)	1.41 [1.01, 1.97]	0.044
Ferritin ≥ 700 µg/L (n = 640)	1.09 [0.77, 1.53]	0.64
D-dimer ≥ 1.5 µg/mL (n = 641)	1.45 [1.07, 1.94]	0.015
Hemoglobin ≤ 9.2 g/dL (n = 982)	2.03 [1.53, 2.69]	<0.005
Neutrophil/Lymphocyte Ratio ≥ 10 (n = 982)	1.29 [1.00, 1.66]	0.050
B-type Natriuretic Peptide ≥ 100 pg/mL (n = 212)	0.88 [0.47, 1.65]	0.69
Troponin I > 0.20 ng/mL or Troponin T > 0.05 ng/mL (n = 876)	1.61 [0.79, 3.28]	0.19
LVEF <50% (n = 212)	2.71 [1.40, 5.25]	<0.005

## Data Availability

The datasets used are available from the corresponding author upon reasonable request.

## Supplementary Materials

The following supporting information can be downloaded at: https://www.mdpi.com/article/10.3390/diagnostics16010038/s1, Supplementary Table S1. ICD-10 Codes; Supplementary Table S2. Cox-proportional and Fine-Gray subdistribution adjusted hazard ratios (HR); Supplementary Table S3. (A) Characteristics of cohort before inverse probability weighting and (B) characteristics of pseudo-population after inverse probability weighting; Appendix S1: Sensitivity analysis without excluding patients who died during acute COVID-19.
