# Supplementary material for: Long-Term Cardiovascular and Mortality Risk in Patients with Pre-Existing Arrhythmia Post-SARS-CoV-2 Infection
Source: Diagnostics (Basel). 2025 Dec 22;16(1):38. doi: 10.3390/diagnostics16010038 (PMC12786083; doi:10.3390/diagnostics16010038)
Supplement: Supplementary file 1 [file diagnostics-16-00038-s001.zip › Arrhythmia Appendix S1.pdf]

Appendix 1

Sensitivity analysis without excluding patients who died.

Figure A.

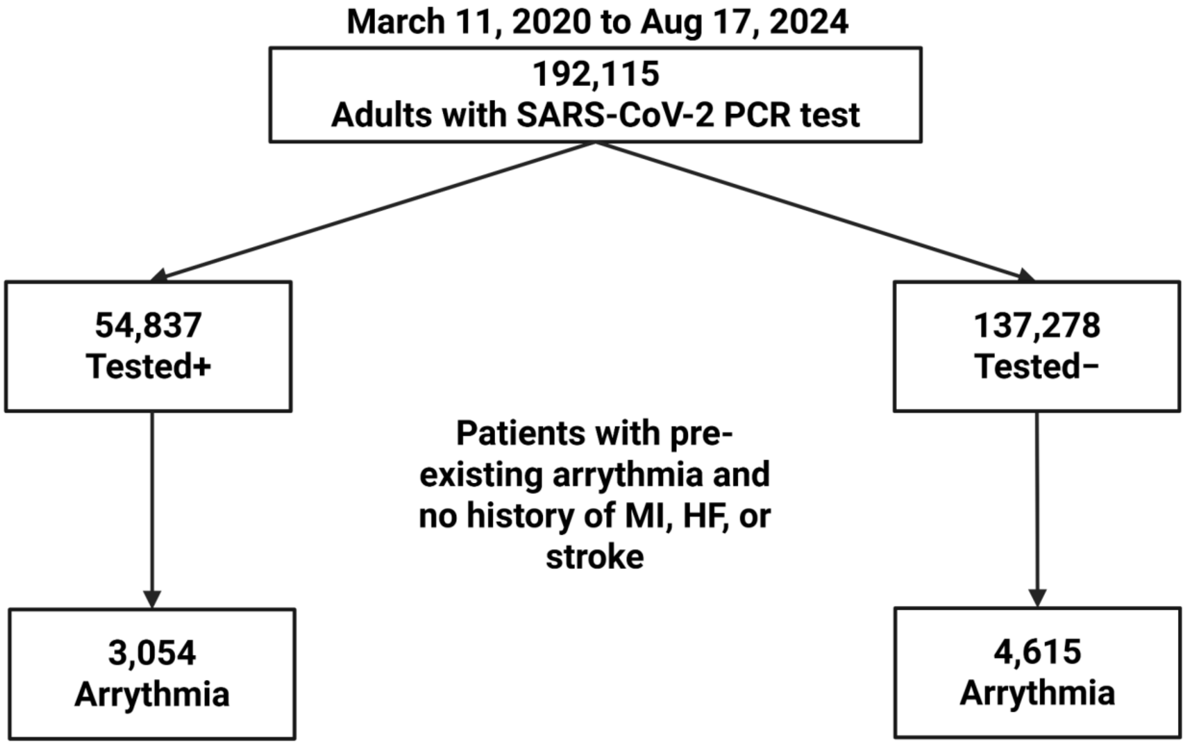

Table A.

	COVID+ (n=3054)	COVID- (n=4615)	*p*-value	SMD
Follow Up Time (Months), mean ± SD	20.23 ± 15.17	26.76 ± 15.55	**<0.005**	0.43
Age at Index Date (Years), mean ± SD	64.28 ± 17.30	60.65 ± 16.82	**<0.005**	0.21
Female, n (%)	1764 (57.76%)	2678 (58.03%)	0.83	0.0054
**Race and Ethnicity, n (%)**				
Non-Hispanic White	416 (13.62%)	698 (15.12%)	0.073	0.043
Black	1041 (34.09%)	1599 (34.65%)	0.63	0.012
Asian	103 (3.37%)	142 (3.08%)	0.51	0.017
Other Race	1494 (48.92%)	2176 (47.15%)	0.14	0.035
Hispanic	1279 (41.88%)	1807 (39.15%)	**0.018**	0.056
**Pre-Existing Comorbidities, n (%)**				
Coronary Artery Disease	647 (21.19%)	712 (15.43%)	**<0.005**	0.15
Hypertension	2306 (75.51%)	3096 (67.09%)	**<0.005**	0.19
Type-2 Diabetes	1239 (40.57%)	1382 (29.95%)	**<0.005**	0.22
COPD	247 (8.09%)	198 (4.29%)	**<0.005**	0.16
Asthma	759 (24.85%)	949 (20.56%)	**<0.005**	0.10
Chronic Kidney Disease	841 (27.54%)	762 (16.51%)	**<0.005**	0.27
Liver Disease	429 (14.05%)	526 (11.40%)	**<0.005**	0.08
Obesity	1722 (56.39%)	2413 (52.29%)	**<0.005**	0.082
Tobacco Use	1235 (40.44%)	1897 (41.11%)	0.58	0.014
**Insurance, n (%)**				
Medicaid	827 (27.08%)	1350 (29.25%)	**0.041**	0.048
Medicare	1079 (35.33%)	1273 (27.58%)	**<0.005**	0.17
Private	998 (32.68%)	1763 (38.20%)	**<0.005**	0.12
Uninsured	150 (4.91%)	229 (4.96%)	0.96	0.0023
**Income Group, n (%)**				
Ischemic or Hemorrhagic Stroke	81 (2.65%)	127 (2.75%)	0.85	0.0061
Major Adverse Cardiovascular Events	663 (21.71%)	609 (13.20%)	**<0.005**	0.23

Table B.

Outcome	A) Multivariate Regression			
	COVID+ Hospitalized vs COVID–		COVID+ Non-Hospitalized vs COVID–	
	Adjusted HR [95% CI]	*p*-value	Adjusted HR [95% CI]	*p*-value
All-Cause Mortality	6.71 [5.25, 8.57]	**<0.005**	2.30 [1.69, 3.13]	**<0.005**
Myocardial Infarction	1.58 [1.16, 2.16]	**<0.005**	0.85 [0.58, 1.26]	0.42
Heart Failure	1.50 [1.24, 1.81]	**<0.005**	0.94 [0.76, 1.16]	0.55
Ischemic or Hemorrhagic Stroke	1.26 [0.89, 1.79]	0.20	0.90 [0.61, 1.32]	0.58
Major Adverse Cardiovascular Events	2.38 [2.09, 2.70]	**<0.005**	1.11 [0.95, 1.30]	0.19
	**B) Inverse Probability Weighting-Adjusted**			
**Outcome**	COVID+ Hospitalized vs COVID–		COVID+ Non-Hospitalized vs COVID–	
	HR [95% CI]	*p*-value	HR [95% CI]	*p*-value
All-Cause Mortality	6.30 [4.90, 8.11]	**<0.005**	2.35 [1.72, 3.21]	**<0.005**
Myocardial Infarction	1.64 [1.14, 2.36]	**<0.005**	0.78 [0.52, 1.17]	0.23
Heart Failure	1.50 [1.21, 1.87]	**<0.005**	0.91 [0.73, 1.13]	0.39
Ischemic or Hemorrhagic Stroke	1.14 [0.78, 1.65]	0.50	0.80 [0.54, 1.18]	0.26
Major Adverse Cardiovascular Events	2.27 [1.95, 2.64]	**<0.005**	1.09 [0.93, 1.29]	0.29
