# Supplementary material for: Long-Term Cardiovascular and Mortality Risk in Patients with Pre-Existing Arrhythmia Post-SARS-CoV-2 Infection
Source: Diagnostics (Basel). 2025 Dec 22;16(1):38. doi: 10.3390/diagnostics16010038 (PMC12786083; doi:10.3390/diagnostics16010038)
Supplement: Supplementary file 1 [file diagnostics-16-00038-s001.zip › Arrhythmia Supplementary Table S1.pdf]

Supplementary Table S1. ICD-10 Codes for outcomes (MI, HF, Stroke, MACE) and arrhythmia subtypes.

HF	MI	Stroke	MACE	Arrhythmia	Atrial Fibrillation	Atrial Flutter	Conduction Disease	Ventricular Arrhythmia	Bradyarrhythmia	SVT	Nonspecific / Miscellaneous Arrhythmia
316139	312327	197303	443454	313217	313217	314665	313791	437894	443522	4275423	44784217
319835	314666	372654	4108356	313791	4141360	36712986	314059	4103295	4169095		
439696	4108218	432923	4110190	314059	4154290	36714994	314379	4111552	4261842		
439846	4108677	443454	4110192	314379	4232691		315069				
443580	4296653	443465	4111714	314665	4232697		316135				
443587	4270024	443525	4112026	315069	45768480		316998				
444101	4329847	443551	45767658	316135			318448				
4004279	37309626	443599	45772786	316998			320425				
4014159	43020460	443609	46270031	318448			320744				
4195785	46270162	4045737	46273649	320425							
4229440	46270163	4045738	260841	320744							
4233424		4046360	432923	437894							
4242669		4108356	4071066	443522							
37309625		4110189	4108952	4103295							
40479192		4110190	4111708	4111552							
40479576		4110192	4111720	4141360							
40480602		4111708	4134162	4154290							
40480603		4111710	4148906	4169095							
40481042		4111711	4110186	4232691							
40481043		4111714	4111721	4232697							
44782718		4111720	4345688	4261842							
44782719		4111721	43530674	4275423							
44782733		4112026	43530727	36712986							
		4134162	312327	36714994							
		4144154	314666	44784217							
		4148906	438172	45768480							
		4159152	4108215								
		4190891	4108218								
		4219010	4108219								
		45767658	4108220								
		45772786	4108677								
		46270031	4108678								
		46273649	4108679								
			4108680								
			4270024								

			4296653								
			4329847								
			37309626								
			37311078								
			43020460								
			45766114								
			45766241								
			46270162								
			46270163								
			319034								
			319835								
			4229440								
			4242669								
			37309625								
			316139								
			319034								
			319835								
			439696								
			439846								
			443580								
			443587								
			444101								
			4004279								
			4014159								
			4195785								
			4229440								
			4233424								
			4242669								
			37309625								
			40479192								
			40479576								
			40480602								
			40480603								
			40481042								
			40481043								
			44782718								
			44782719								
			44782733								

			198571								
			321042								
			4106274								
			4172822								
			4309332								
			4311273								
			37398951								
			46269812								
			46274066								
			314659								
			320749								
			380747								
			4290976								
			4343935								
			381591								
			434056								
			40479575								
			40480002								
			40480938								
			40481842								
			43530687								
			43530688								
			43531622								
			372547								
			373408								
			373692								
			376026								
			376905								
			377487								
			378065								
			380632								
			434866								
			440023								
			4041669								
			4045976								
			4045978								
			4103105								
			4147498								

			4150516								
			4210628								
			4249574								
			40479557								
			46269919								
			312938								
			372892								
			373764								
			377845								
			433957								
			4027461								
			4190891								
			4219010								
			4252885								
			4318859								
			42872891								
			432738								
			442024								
			4202045								
			4223659								
			432556								
			432879								
			433403								
			434860								
			435182								
			435785								
			436090								
			436091								
			436903								
			437538								
			437772								
			438338								
			438418								
			439797								
			440323								
			440390								
			440699								
			441787								

			4044930								
			4044931								
			4047472								
			4102297								
			4105194								
			4136344								
			4207307								
			4219348								
			46269917								
			374168								
			377091								
			377101								
			4101747								
			4194232								
			4233724								
			4236312								
			43530626								
			43531630								
			46270364								
