# Supplementary material for: Long-Term Cardiovascular and Mortality Risk in Patients with Pre-Existing Arrhythmia Post-SARS-CoV-2 Infection
Source: Diagnostics (Basel). 2025 Dec 22;16(1):38. doi: 10.3390/diagnostics16010038 (PMC12786083; doi:10.3390/diagnostics16010038)
Supplement: Supplementary file 1 [file diagnostics-16-00038-s001.zip › Arrhythmia Supplementary Table S2.pdf]

Supplementary Table S2. Cox-proportional (all-cause mortality and major adverse cardiovascular events) and Fine-Gray subdistribution (myocardial infarction, congestive heart failure, and ischemic or hemorrhagic stroke) adjusted hazard ratios (HR) for different outcomes grouped by COVID-19 status (COVID+ hospitalized and COVID+ non-hospitalized versus COVID– controls). Multivariate models were adjusted for baseline age, sex, race, ethnicity, comorbidities, insurance status, tertile of Zone Improvement Plan median income, presence of unmet social needs, and SARS-CoV-2 vaccination status. HR, hazard ratio. CI, confidence interval.

**All-Cause Mortality**				
**Covariate**	Unadjusted HR [95% CI]	*p*-value	Adjusted HR [95% CI]	*p*-value
**COVID-19 Status**				
COVID+ Hospitalized vs COVID–	4.21 [3.15, 5.61]	**<0.005**	2.90 [2.08, 4.04]	**<0.005**
COVID+ Non-Hospitalized vs COVID–	0.96 [0.67, 1.36]	0.80	1.67 [1.13, 2.47]	**0.010**
Vaccination for SARS-CoV-2	0.92 [0.67, 1.27]	0.62	0.91 [0.65, 1.26]	0.57
**Age and Sex**				
Age at Index Date (Years)	1.05 [1.04, 1.07]	**<0.005**	1.04 [1.03, 1.06]	**<0.005**
Male vs Female	1.39 [1.05, 1.84]	**0.022**	1.23 [0.91, 1.65]	0.18
**Race and Ethnicity**				
Black vs Non-Hispanic White	0.89 [0.66, 1.21]	0.47	0.78 [0.51, 1.19]	0.25
Asian vs Non-Hispanic White	0.47 [0.15, 1.48]	0.20	0.58 [0.18, 1.90]	0.37
Other Race vs Non-Hispanic White	0.93 [0.70, 1.23]	0.61	0.92 [0.54, 1.57]	0.75
Hispanic vs Non-Hispanic	0.88 [0.66, 1.17]	0.38	0.94 [0.59, 1.52]	0.81
**Pre-Existing Comorbidities**				
Coronary Artery Disease	1.58 [1.14, 2.19]	**0.0060**	0.80 [0.57, 1.13]	0.21
Hypertension	2.78 [1.83, 4.23]	**<0.005**	1.00 [0.62, 1.60]	0.99
Type-2 Diabetes	2.06 [1.56, 2.73]	**<0.005**	1.20 [0.88, 1.63]	0.24
COPD	3.38 [2.31, 4.94]	**<0.005**	1.98 [1.31, 2.98]	**<0.005**
Asthma	1.06 [0.76, 1.47]	0.73	1.12 [0.79, 1.58]	0.52
Chronic Kidney Disease	3.29 [2.48, 4.37]	**<0.005**	1.88 [1.38, 2.57]	**<0.005**
Liver Disease	2.09 [1.50, 2.91]	**<0.005**	1.84 [1.31, 2.59]	**<0.005**
Tobacco Use	1.28 [0.96, 1.69]	0.091	1.01 [0.75, 1.36]	0.94
Obesity	0.90 [0.68, 1.19]	0.45	1.03 [0.77, 1.39]	0.84
**Insurance**				
Medicaid vs Private Insurance	0.74 [0.53, 1.04]	**0.080**	1.78 [1.15, 2.73]	**0.0090**
Medicare vs Private Insurance	2.36 [1.78, 3.13]	**<0.005**	1.29 [0.90, 1.86]	0.17
Uninsured vs Private Insurance	0.52 [0.21, 1.27]	0.15	1.13 [0.44, 2.90]	0.80
**Income Tertile**				
Lower Third vs Top Third	0.77 [0.57, 1.03]	0.081	0.62 [0.43, 0.88]	**0.0070**
Middle Third vs Top Third	0.88 [0.64, 1.21]	0.45	0.66 [0.46, 0.94]	**0.021**
**Unmet Social Needs**				
At Least One Unmet Social Need vs None	0.50 [0.27, 0.91]	**0.024**	0.75 [0.38, 1.46]	0.40

Myocardial Infarction				
**Covariate**	Unadjusted HR [95% CI]	*p*-value	Adjusted HR [95% CI]	*p*-value
**COVID-19 Status**				
COVID+ Hospitalized vs COVID–	2.67 [1.99, 3.59]	**<0.005**	1.57 [1.14, 2.17]	**0.0060**
COVID+ Non-Hospitalized vs COVID–	0.66 [0.45, 0.98]	**0.037**	0.82 [0.55, 1.23]	0.34
Vaccination for SARS-CoV-2	1.33 [0.99, 1.80]	0.060	1.39 [1.03, 1.89]	**0.033**
**Age and Sex**				
Age at Index Date (Years)	1.05 [1.04, 1.06]	**<0.005**	1.04 [1.02, 1.05]	**<0.005**
Male vs Female	1.18 [0.90, 1.55]	0.22	1.13 [0.85, 1.51]	0.40
**Race and Ethnicity**				
Black vs Non-Hispanic White	1.23 [0.94, 1.62]	0.13	1.52 [0.96, 2.38]	0.071
Asian vs Non-Hispanic White	0.58 [0.22, 1.56]	0.28	0.79 [0.27, 2.31]	0.67
Other Race vs Non-Hispanic White	0.88 [0.67, 1.15]	0.36	0.86 [0.48, 1.55]	0.62
Hispanic vs Non-Hispanic	1.02 [0.78, 1.34]	0.88	1.51 [0.94, 2.42]	0.089
**Pre-Existing Comorbidities**				
Coronary Artery Disease	3.17 [2.41, 4.17]	**<0.005**	2.01 [1.50, 2.70]	**<0.005**
Hypertension	4.31 [2.69, 6.90]	**<0.005**	1.81 [1.08, 3.02]	**0.024**
Type-2 Diabetes	1.83 [1.40, 2.39]	**<0.005**	0.99 [0.74, 1.32]	0.94
COPD	2.18 [1.43, 3.34]	**<0.005**	1.15 [0.73, 1.80]	0.55
Asthma	1.30 [0.97, 1.75]	0.081	1.39 [1.01, 1.90]	**0.041**
Chronic Kidney Disease	2.47 [1.88, 3.25]	**<0.005**	1.39 [1.03, 1.88]	**0.034**
Liver Disease	0.79 [0.51, 1.22]	0.28	0.63 [0.40, 0.97]	**0.037**
Tobacco Use	1.50 [1.15, 1.96]	**<0.005**	1.30 [0.98, 1.73]	0.064
Obesity	0.94 [0.72, 1.24]	0.68	0.96 [0.72, 1.27]	0.77
**Insurance**				
Medicaid vs Private Insurance	0.67 [0.49, 0.94]	**0.019**	1.55 [1.03, 2.33]	**0.037**
Medicare vs Private Insurance	2.22 [1.70, 2.90]	**<0.005**	1.24 [0.88, 1.74]	0.22
Uninsured vs Private Insurance	0.72 [0.35, 1.45]	0.35	2.05 [0.95, 4.39]	0.066
**Income Tertile**				
Lower Third vs Top Third	0.96 [0.73, 1.26]	0.76	1.08 [0.76, 1.55]	0.66
Middle Third vs Top Third	1.27 [0.96, 1.68]	0.093	1.26 [0.89, 1.79]	0.20
**Unmet Social Needs**				
At Least One Unmet Social Need vs None	0.78 [0.49, 1.25]	0.30	1.09 [0.64, 1.86]	0.74

Heart Failure				
**Covariate**	Unadjusted HR [95% CI]	*p*-value	Adjusted HR [95% CI]	*p*-value
**COVID-19 Status**				
COVID+ Hospitalized vs COVID–	2.38 [1.99, 2.86]	**<0.005**	1.51 [1.24, 1.84]	**<0.005**
COVID+ Non-Hospitalized vs COVID–	0.78 [0.63, 0.97]	**0.024**	0.94 [0.75, 1.18]	0.62
Vaccination for SARS-CoV-2	1.12 [0.93, 1.33]	0.23	1.08 [0.90, 1.29]	0.43
**Age and Sex**				
Age at Index Date (Years)	1.04 [1.04, 1.05]	**<0.005**	1.03 [1.02, 1.04]	**<0.005**
Male vs Female	1.22 [1.04, 1.43]	**0.014**	1.17 [0.99, 1.39]	0.067
**Race and Ethnicity**				
Black vs Non-Hispanic White	1.03 [0.88, 1.22]	0.71	0.95 [0.73, 1.22]	0.67
Asian vs Non-Hispanic White	0.59 [0.34, 1.05]	0.074	0.74 [0.40, 1.36]	0.33
Other Race vs Non-Hispanic White	0.96 [0.82, 1.13]	0.64	0.88 [0.63, 1.22]	0.44
Hispanic vs Non-Hispanic	0.99 [0.85, 1.17]	0.94	1.06 [0.81, 1.38]	0.69
**Pre-Existing Comorbidities**				
Coronary Artery Disease	2.27 [1.92, 2.70]	**<0.005**	1.34 [1.12, 1.61]	**<0.005**
Hypertension	4.48 [3.39, 5.93]	**<0.005**	2.01 [1.48, 2.73]	**<0.005**
Type-2 Diabetes	2.08 [1.78, 2.44]	**<0.005**	1.18 [1.00, 1.40]	0.052
COPD	2.23 [1.74, 2.88]	**<0.005**	1.35 [1.03, 1.76]	**0.028**
Asthma	1.07 [0.89, 1.28]	0.48	1.05 [0.87, 1.28]	0.59
Chronic Kidney Disease	2.52 [2.14, 2.96]	**<0.005**	1.43 [1.20, 1.70]	**<0.005**
Liver Disease	1.12 [0.89, 1.40]	0.33	0.88 [0.70, 1.11]	0.28
Tobacco Use	1.42 [1.21, 1.66]	**<0.005**	1.19 [1.00, 1.40]	**0.044**
Obesity	1.18 [1.00, 1.38]	**0.047**	1.19 [1.01, 1.41]	**0.042**
**Insurance**				
Medicaid vs Private Insurance	0.63 [0.52, 0.77]	**<0.005**	1.15 [0.90, 1.46]	0.26
Medicare vs Private Insurance	2.24 [1.91, 2.62]	**<0.005**	1.16 [0.95, 1.42]	0.13
Uninsured vs Private Insurance	0.30 [0.16, 0.56]	**<0.005**	0.67 [0.35, 1.28]	0.23
**Income Tertile**				
Lower Third vs Top Third	1.20 [1.02, 1.40]	**0.028**	1.28 [1.04, 1.58]	**0.019**
Middle Third vs Top Third	1.02 [0.86, 1.21]	0.83	1.11 [0.90, 1.38]	0.33
**Unmet Social Needs**				
At Least One Unmet Social Need vs None	0.98 [0.76, 1.27]	0.90	1.07 [0.80, 1.42]	0.65

Ischemic or Hemorrhagic Stroke				
**Covariate**	Unadjusted HR [95% CI]	*p*-value	Adjusted HR [95% CI]	*p*-value
**COVID-19 Status**				
COVID+ Hospitalized vs COVID–	1.83 [1.31, 2.56]	**<0.005**	1.24 [0.87, 1.78]	0.24
COVID+ Non-Hospitalized vs COVID–	0.73 [0.50, 1.08]	0.12	0.83 [0.55, 1.24]	0.36
Vaccination for SARS-CoV-2	1.12 [0.81, 1.55]	0.48	1.04 [0.75, 1.45]	0.79
**Age and Sex**				
Age at Index Date (Years)	1.04 [1.03, 1.05]	**<0.005**	1.03 [1.02, 1.04]	**<0.005**
Male vs Female	0.98 [0.74, 1.31]	0.89	0.96 [0.71, 1.30]	0.79
**Race and Ethnicity**				
Black vs Non-Hispanic White	1.25 [0.94, 1.67]	0.12	1.11 [0.70, 1.78]	0.65
Asian vs Non-Hispanic White	1.64 [0.87, 3.10]	0.13	1.93 [0.91, 4.10]	0.086
Other Race vs Non-Hispanic White	0.75 [0.57, 1.00]	0.05	0.79 [0.43, 1.45]	0.44
Hispanic vs Non-Hispanic	0.86 [0.64, 1.15]	0.30	1.15 [0.70, 1.87]	0.58
**Pre-Existing Comorbidities**				
Coronary Artery Disease	1.80 [1.31, 2.47]	**<0.005**	1.14 [0.82, 1.59]	0.44
Hypertension	3.47 [2.20, 5.45]	**<0.005**	1.72 [1.04, 2.83]	**0.034**
Type-2 Diabetes	1.94 [1.47, 2.57]	**<0.005**	1.24 [0.92, 1.67]	0.17
COPD	1.66 [1.01, 2.73]	**0.046**	0.98 [0.58, 1.66]	0.95
Asthma	1.20 [0.87, 1.64]	0.27	1.15 [0.83, 1.61]	0.40
Chronic Kidney Disease	2.44 [1.83, 3.25]	**<0.005**	1.46 [1.06, 2.01]	**0.019**
Liver Disease	1.01 [0.67, 1.53]	0.95	0.82 [0.54, 1.24]	0.34
Tobacco Use	1.45 [1.09, 1.91]	**0.010**	1.36 [1.01, 1.82]	**0.041**
Obesity	1.01 [0.76, 1.34]	0.93	0.97 [0.72, 1.31]	0.84
**Insurance**				
Medicaid vs Private Insurance	0.65 [0.46, 0.92]	**0.014**	1.10 [0.72, 1.67]	0.67
Medicare vs Private Insurance	1.84 [1.38, 2.44]	**<0.005**	1.02 [0.72, 1.45]	0.91
Uninsured vs Private Insurance	0.58 [0.26, 1.31]	0.19	1.11 [0.47, 2.63]	0.81
**Income Tertile**				
Lower Third vs Top Third	1.06 [0.80, 1.41]	0.70	1.15 [0.80, 1.67]	0.45
Middle Third vs Top Third	1.11 [0.82, 1.50]	0.48	1.13 [0.78, 1.63]	0.53
**Unmet Social Needs**				
At Least One Unmet Social Need vs None	1.18 [0.78, 1.80]	0.43	1.10 [0.69, 1.75]	0.69

Major Adverse Cardiovascular Events				
**Covariate**	Unadjusted HR [95% CI]	*p*-value	Adjusted HR [95% CI]	*p*-value
**COVID-19 Status**				
COVID+ Hospitalized vs COVID–	2.52 [2.18, 2.90]	**<0.005**	1.64 [1.40, 1.91]	**<0.005**
COVID+ Non-Hospitalized vs COVID–	0.80 [0.68, 0.94]	**0.0080**	0.99 [0.83, 1.18]	0.92
Vaccination for SARS-CoV-2	1.10 [0.95, 1.26]	0.19	1.06 [0.92, 1.23]	0.40
**Age and Sex**				
Age at Index Date (Years)	1.04 [1.04, 1.05]	**<0.005**	1.03 [1.02, 1.03]	**<0.005**
Male vs Female	1.22 [1.07, 1.38]	**<0.005**	1.14 [1.00, 1.31]	**0.046**
**Race and Ethnicity**				
Black vs Non-Hispanic White	1.05 [0.92, 1.20]	0.45	0.98 [0.81, 1.20]	0.88
Asian vs Non-Hispanic White	0.81 [0.55, 1.20]	0.29	0.96 [0.63, 1.46]	0.84
Other Race vs Non-Hispanic White	0.91 [0.80, 1.03]	0.12	0.86 [0.66, 1.11]	0.24
Hispanic vs Non-Hispanic	0.95 [0.84, 1.08]	0.47	1.10 [0.89, 1.37]	0.37
**Pre-Existing Comorbidities**				
Coronary Artery Disease	2.28 [1.99, 2.61]	**<0.005**	1.37 [1.18, 1.58]	**<0.005**
Hypertension	3.68 [3.00, 4.50]	**<0.005**	1.72 [1.37, 2.14]	**<0.005**
Type-2 Diabetes	1.94 [1.72, 2.20]	**<0.005**	1.12 [0.98, 1.28]	0.10
COPD	2.31 [1.89, 2.81]	**<0.005**	1.37 [1.11, 1.69]	**<0.005**
Asthma	1.10 [0.95, 1.27]	0.19	1.10 [0.95, 1.28]	0.20
Chronic Kidney Disease	2.55 [2.24, 2.90]	**<0.005**	1.48 [1.29, 1.71]	**<0.005**
Liver Disease	1.23 [1.03, 1.46]	**0.019**	0.97 [0.82, 1.16]	0.76
Tobacco Use	1.39 [1.23, 1.58]	**<0.005**	1.18 [1.04, 1.35]	**0.013**
Obesity	1.09 [0.96, 1.24]	0.18	1.11 [0.97, 1.27]	0.12
**Insurance**				
Medicaid vs Private Insurance	0.69 [0.60, 0.81]	**<0.005**	1.27 [1.06, 1.53]	**0.011**
Medicare vs Private Insurance	2.07 [1.83, 2.35]	**<0.005**	1.14 [0.97, 1.33]	0.11
Uninsured vs Private Insurance	0.45 [0.30, 0.67]	**<0.005**	0.94 [0.62, 1.44]	0.78
**Income Tertile**				
Lower Third vs Top Third	1.05 [0.92, 1.19]	0.49	1.09 [0.92, 1.28]	0.32
Middle Third vs Top Third	1.06 [0.92, 1.21]	0.42	1.05 [0.89, 1.24]	0.55
**Unmet Social Needs**				
At Least One Unmet Social Need vs None	0.94 [0.76, 1.15]	0.54	1.04 [0.82, 1.31]	0.75
