# Supplementary material for: Long-Term Cardiovascular and Mortality Risk in Patients with Pre-Existing Arrhythmia Post-SARS-CoV-2 Infection
Source: Diagnostics (Basel). 2025 Dec 22;16(1):38. doi: 10.3390/diagnostics16010038 (PMC12786083; doi:10.3390/diagnostics16010038)
Supplement: Supplementary file 1 [file diagnostics-16-00038-s001.zip › Arrhythmia Supplementary Table S3.pdf]

Supplementary Table S3. A) Characteristics of cohort before inverse probability weighting and **B)** characteristics of pseudo-population after inverse probability weighting. SD, standard deviation. SMD, standardized mean difference. COPD, chronic obstructive pulmonary disease.

**A)**	**COVID+ Hospitalized (n=985)**	**COVID+ Non-Hospitalized (n=1591)**	**COVID− (n=4254)**	**COVID+ Hospitalized vs COVID−**		**COVID+ Non-Hospitalized vs COVID−**	
				***p*-value**	**SMD**	***p*-value**	**SMD**
Follow Up Time (Months), mean ± SD	25.06 ± 15.59	22.80 ± 12.48	28.81 ± 14.23	**<0.005**	0.25	**<0.005**	0.45
Age at Index Date (Years), mean ± SD	68.71 ± 16.43	60.25 ± 16.68	60.74 ± 16.63	**<0.005**	0.48	0.31	0.030
Female, n (%)	544 (55.23%)	1000 (62.85%)	2506 (58.91%)	**0.038**	0.074	**0.0067**	0.081
**Race and Ethnicity, n (%)**							
Non-Hispanic White	131 (13.30%)	196 (12.32%)	631 (14.83%)	0.24	0.044	**0.016**	0.073
Black	354 (35.94%)	536 (33.69%)	1481 (34.81%)	0.53	0.024	0.44	0.024
Asian	27 (2.74%)	60 (3.77%)	133 (3.13%)	0.60	0.023	0.25	0.035
Other Race	473 (48.02%)	799 (50.22%)	2009 (47.23%)	0.68	0.016	**0.044**	0.060
Hispanic	411 (41.73%)	689 (43.31%)	1682 (39.54%)	0.22	0.045	**0.0099**	0.077
**Pre-Existing Comorbidities, n (%)**							
Arrhythmia	985 (100.00%)	1591 (100.00%)	4254 (100.00%)		0		0
Atrial Fibrillation	346 (35.13%)	369 (23.19%)	983 (23.11%)	**<0.005**	0.27	0.97	0.002
Atrial Flutter	78 (7.92%)	63 (3.96%)	145 (3.41%)	**<0.005**	0.2	0.35	0.029
Conduction Disease	343 (34.82%)	392 (24.64%)	1089 (25.60%)	**<0.005**	0.2	0.47	0.022
Ventricular Arrhythmia	66 (6.70%	79 (4.97%	187 (4.40%)	**<0.005**	0.1	0.39	0.027
Bradyarrhythmia	354 (35.94%)	647 (40.67%)	1728 (40.62%)	0.0076	0.096	1	0.00093
SVT	122 (12.39%)	158 (9.93%)	331 (7.78%)	**<0.005**	0.15	0.0096	0.076
Nonspecific/Miscellaneous Arrhythmia	155 (15.74%)	276 (17.35%)	649 (15.26%)	0.74	0.013	0.056	0.057
Coronary Artery Disease	271 (27.51%)	262 (16.47%)	659 (15.49%)	**<0.005**	0.30	0.38	0.027
Hypertension	850 (86.29%)	1095 (68.82%)	2909 (68.38%)	**<0.005**	0.44	0.77	0.0095
Type-2 Diabetes	503 (51.07%)	526 (33.06%)	1297 (30.49%)	**<0.005**	0.43	0.063	0.055
COPD	122 (12.39%)	79 (4.97%)	189 (4.44%)	**<0.005**	0.29	0.44	0.025
Asthma	246 (24.97%)	435 (27.34%)	906 (21.30%)	**0.014**	0.087	**<0.005**	0.14
Chronic Kidney Disease	366 (37.16%)	330 (20.74%)	724 (17.02%)	**<0.005**	0.47	**<0.005**	0.095
Liver Disease	168 (17.06%)	207 (13.01%)	505 (11.87%)	**<0.005**	0.15	0.25	0.035
Obesity	556 (56.45%)	946 (59.46%)	2274 (53.46%)	0.097	0.06	**<0.005**	0.12
Tobacco Use	441 (44.77%)	626 (39.35%)	1751 (41.16%)	**0.042**	0.073	0.22	0.037
**Insurance, n (%)**							
Medicaid	254 (25.79%)	464 (29.16%)	1231 (28.94%)	0.053	0.071	0.89	0.0050
Medicare	434 (44.06%)	412 (25.90%)	1167 (27.43%)	**<0.005**	0.35	0.25	0.035
Private	277 (28.12%)	617 (38.78%)	1649 (38.76%)	**<0.005**	0.23	1.00	0.00035
Uninsured	20 (2.03%)	98 (6.16%)	207 (4.87%)	**<0.005**	0.16	0.056	0.057
**Annual Income Group, n (%)**							
Lower Third (≤$42,639)	400 (40.61%)	625 (39.28%)	1636 (38.46%)	0.23	0.044	0.58	0.017
Middle Third ($42,834–$61,272)	310 (31.47%)	493 (30.99%)	1234 (29.01%)	0.14	0.054	0.15	0.043
Top Third (≥$61,414)	275 (27.92%)	473 (29.73%)	1384 (32.53%)	**0.0056**	0.10	**0.044**	0.061
**Unmet Social Needs, n (%)**							
At Least One Unmet Social Need	84 (8.53%)	185 (11.63%)	424 (9.97%)	0.19	0.05	0.072	0.054
No Unmet Social Needs	255 (25.89%)	535 (33.63%)	1239 (29.13%)	**0.047**	0.073	**<0.005**	0.097
Status Unknown	646 (65.58%)	871 (54.75%)	2591 (60.91%)	**0.0072**	0.097	**<0.005**	0.13
**Vaccinated for SARS-CoV-2, n (%)**	288 (29.24%)	760 (47.77%)	1298 (30.51%)	0.46	0.028	**<0.005**	0.36
**Outcomes, n (%)**							
All-Cause Mortality	77 (7.82%)	39 (2.45%)	78 (1.83%)	**<0.005**	0.28	0.16	0.043
Myocardial Infarction	63 (6.40%)	30 (1.89%)	122 (2.87%)	**<0.005**	0.17	**0.045**	0.065
Heart Failure	159 (16.14%)	101 (6.35%)	356 (8.37%)	**<0.005**	0.24	**0.012**	0.077
Ischemic or Hemorrhagic Stroke	44 (4.47%)	30 (1.89%)	122 (2.87%)	**0.013**	0.085	**0.045**	0.065
Major Adverse Cardiovascular Events	265 (26.90%)	168 (10.56%)	553 (13.00%)	**<0.005**	0.35	**0.013**	0.076

B)	COVID+ Hospitalized (n=988)	COVID+ Non-Hospitalized (n=1595)	COVID− (n=4250)	COVID+ Hospitalized vs COVID−		COVID+ Non-Hospitalized vs COVID−	
				p-value	SMD	*p*-value	SMD
Follow Up Time (Months), mean ± SD	25.48 ± 15.30	23.35 ± 13.03	28.51 ± 14.19	**<0.005**	0.21	**<0.005**	0.38
Age at Index Date (Years), mean ± SD	60.69 ± 18.89	61.80 ± 16.70	61.78 ± 16.59	**<0.005**	0.061	0.31	0.00087
Female, n (%)	593 (60.03%)	945 (59.28%)	2529 (59.51%)	0.79	0.011	0.9	0.0046
**Race and Ethnicity, n (%)**							
Non-Hispanic White	128 (13.04%)	232 (14.59%)	596 (14.03%)	0.45	0.029	0.61	0.016
Black	341 (34.54%)	542 (34.03%)	1475 (34.72%)	0.94	0.0039	0.64	0.015
Asian	29 (2.99%)	53 (3.35%)	137 (3.23%)	0.77	0.014	0.89	0.0065
Other Race	488 (49.43%)	766 (48.03%)	2040 (48.02%)	0.44	0.028	1.00	0.00035
Hispanic	417 (42.24%)	648 (40.67%)	1731 (40.74%)	0.41	0.031	0.99	0.0013
**Pre-Existing Comorbidities, n (%)**							
Arrhythmia	988 (100.00%)	1595 (100.00%)	4250 (100.00%)		0		0
Atrial Fibrillation	292 (29.62%)	388 (24.38%)	1004 (23.63%)	**<0.005**	0.14	0.57	0.017
Atrial Flutter	66 (6.75%)	64 (4.02%)	150 (3.55%)	**<0.005**	0.15	0.44	0.025
Conduction Disease	307 (31.10%)	398 (24.97%)	1114 (26.22%)	**<0.005**	0.11	0.35	0.029
Ventricular Arrhythmia	69 (6.98%)	80 (5.04%)	184 (4.34%)	**<0.005**	0.11	0.28	0.033
Bradyarrhythmia	366 (37.08%)	652 (40.89%)	1709 (40.21%)	0.076	0.064	0.66	0.014
SVT	128 (13.00%)	151 (9.50%)	332 (7.82%)	**<0.005**	0.17	0.043	0.06
Nonspecific/Miscellaneous Arrhythmia	144 (14.63%)	262 (16.45%)	649 (15.28%)	0.64	0.018	0.29	0.032
Coronary Artery Disease	176 (17.81%)	279 (17.51%)	740 (17.42%)	0.81	0.01	0.97	0.0024
Hypertension	704 (71.29%)	1136 (71.27%)	3022 (71.10%)	0.94	0.004	0.93	0.0036
Type-2 Diabetes	345 (34.96%)	555 (34.82%)	1448 (34.07%)	0.62	0.019	0.61	0.016
COPD	58 (5.96%)	90 (5.65%)	240 (5.65%)	0.77	0.013	1.00	0.00031
Asthma	255 (25.87%)	361 (22.66%)	987 (23.22%)	0.086	0.061	0.68	0.013
Chronic Kidney Disease	206 (20.91%)	338 (21.19%)	886 (20.85%)	1.00	0.0014	0.80	0.0083
Liver Disease	134 (13.65%)	209 (13.16%)	545 (12.83%)	0.52	0.024	0.77	0.010
Obesity	566 (57.28%)	887 (55.62%)	2355 (55.42%)	0.31	0.038	0.91	0.0040
Tobacco Use	415 (42.02%)	658 (41.27%)	1752 (41.24%)	0.68	0.016	1.00	0.00063
**Insurance, n (%)**							
Medicaid	281 (28.47%)	464 (29.12%)	1210 (28.48%)	1.00	0.00041	0.66	0.014
Medicare	292 (29.63%)	481 (30.19%)	1254 (29.52%)	0.98	0.0024	0.64	0.015
Private	383 (38.78%)	579 (36.32%)	1586 (37.32%)	0.41	0.030	0.50	0.021
Uninsured	30 (3.13%)	69 (4.37%)	199 (4.68%)	0.039	0.080	0.66	0.015
**Annual Income Group, n (%)**							
Lower Third (≤$42,639)	397 (40.22%)	625 (39.22%)	1656 (38.98%)	0.50	0.025	0.89	0.0048
Middle Third ($42,834/–$61,272)	291 (29.48%)	476 (29.88%)	1270 (29.89%)	0.83	0.0092	1.00	0.00025
Top Third (≥$61,414)	299 (30.31%)	492 (30.90%)	1322 (31.12%)	0.65	0.018	0.89	0.0048
**Unmet Social Needs, n (%)**							
At Least One Unmet Social Need	115 (11.67%)	163 (10.24%)	431 (10.15%)	0.18	0.049	0.95	0.0031
No Unmet Social Needs	296 (30.00%)	467 (29.30%)	1260 (29.67%)	0.87	0.0073	0.81	0.0081
Status Unknown	576 (58.33%)	964 (60.46%)	2558 (60.19%)	0.30	0.038	0.87	0.0056
**Vaccinated for SARS-CoV-2, n (%)**	337 (34.10%)	543 (34.05%)	1455 (34.25%)	0.96	0.0032	0.91	0.0043
**Outcomes, n (%)**							
All-Cause Mortality	53 (5.39%)	44 (2.81%)	83 (1.97%)	**<0.005**	0.18	0.062	0.056
Myocardial Infarction	49 (4.97%)	31 (1.95%)	135 (3.18%)	**0.0078**	0.091	**0.015**	0.078
Heart Failure	118 (12.00%)	108 (6.78%)	382 (9.01%)	**<0.005**	0.098	**0.0071**	0.083
Ischemic or Hemorrhagic Stroke	34 (3.45%)	31 (1.94%)	132 (3.11%)	0.66	0.019	**0.020**	0.075
Major Adverse Cardiovascular Events	202 (20.44%)	180 (11.31%)	593 (13.95%)	**<0.005**	0.17	**0.0090**	0.080
